# Medically inoperable peripheral lung cancer treated with stereotactic body radiation therapy

**DOI:** 10.1186/s13014-015-0423-7

**Published:** 2015-05-28

**Authors:** K. D. Kelley, D. L. Benninghoff, J. S. Stein, J. Z. Li, R. T. Byrnes, L. Potters, J. P. S. Knisely, H. D. Zinkin

**Affiliations:** The Department of Radiation Medicine, North Shore-LIJ Cancer Institute, Lake Success, NY USA; The Department of Radiation Medicine, North Shore-LIJ Health System, Huntington, NY USA; The Department of Biostatistics, Feinstein Institute for Medical Research, North Shore-LIJ Health System, Manhasset, NY USA; 989W Jericho Turnpike, Smithtown, NY 11787 USA

## Abstract

**Background:**

Lung cancer is the most frequent cause of cancer-related death in North America. There is wide variation between patients who are medically inoperable and those managed surgically. The use of stereotactic body radiotherapy (SBRT) has narrowed the gap in survival rates between operative and non-operative management for those with early stage disease. This retrospective study reports outcomes for the treatment of peripheral non-small cell lung carcinoma (NSCLC) with SBRT from a single community practice.

**Methods:**

Sixty-seven consecutive patients (pts) with inoperable, untreated peripheral lung tumors were treated from 2010 through 2012 and included in this study. Stereotactic targeting was facilitated by either spine or lung-based image guidance, either with or without fiducial marker tracking with a frameless robotic radiosurgery system. Peripheral tumors received a median biological effective dose (BED) of 105.6 Gy_10_ or in terms of a median physical dose, 48 Gy delivered over 4 daily fractions. Survival was measured using the Kaplan-Meier method to determine rates of local control, progression of disease and overall survival. The Cox proportional hazards regression model was used to study the effects of tumor size, stage, histology, patient age, tumor location (lobe), tracking method, and BED on the survival distributions.

**Results:**

The median follow-up for this cohort was 24.5 months (range: 2.4–50.3) with an overall (OS) 3-year survival of 62.4 % (95 % CI: 74.3-47.3). The median progression-free survival was 28.5 months (95 % CI: 15.8 months to not reached). Local control (LC), defined as a lack of FDG uptake on PET/CT or the absence of tumor growth was achieved in 60 patients (90.9 %) at the time of first follow-up (median 3 months, range: 1–6). Local control at one year for the entire cohort was 81.8 % (95 % CI, 67.3-90.3). The one-year OS probability among those who achieved local control at first follow-up was 86.2 % (95 % CI, 74.3-92.9) but no patients who did not achieve LC at first follow-up survived one year. Of the 60 pts that achieved initial LC, 16 have died. The rates of local control, progression-free survival and overall survival were not statistically different for patients treated using a fiducial target tracking system versus non-invasive guidance. (*p* = 0.44, *p* = 0.97 and *p* = 0.66, respectively). No National Cancer Institute (NCI) Common Terminology Criteria for Adverse Events (CTCAE-4) grade 3 or greater toxicity was observed.

**Conclusion:**

SBRT is an effective treatment for medically inoperable NSCLC patients with peripherally located tumors. This therapy appears to be well tolerated with low toxicity, and patient outcomes when using non-invasive tumor tracking systems are not inferior to traditional fiducial-based techniques.

**Electronic supplementary material:**

The online version of this article (doi:10.1186/s13014-015-0423-7) contains supplementary material, which is available to authorized users.

## Background

Until the advent of stereotactic body radiation therapy (SBRT), patients with early and intermediate stage lung cancer who were medically inoperable had limited treatment options [1]. Traditional protracted radiotherapy yielded relatively poor outcomes for this patient population historically with a 5-year overall survival of only 13–39% [2]. Sophisticated treatment planning and image guidance now permits focal high-dose per fraction radiation to be delivered safely [3, 4]. This approach of dose escalation delivered to a defined target while preserving adjacent healthy tissues with strict geometric avoidance of organs at risk (OARs) is what constitutes SBRT. Clinical trials have shown an improvement of the therapeutic ratio compared to results from conventional radiotherapy with enhanced control rates and low toxicity to OARs [1, 5–11]. This approach has in turn proven very effective in controlling tumors in medically inoperable patients and is now considered a standard of care for this patient population [1].

The purpose of this study was to analyze the outcomes of patients treated with SBRT for early and intermediate stage NSCLC within a community-based setting, in order to outline potential prognostic factors and assess variations in survival and local control rates when using different methods of image guidance. Moreover, data analyzed from a community-based practice may better estimate non-academic centers’ outcomes and be more representative of likely outcomes outside tertiary centers. Additionally, lower dose per fraction regimens, similar to those used in this study and known to carry less risk of inducing acute and long-term toxicities, are hypothesized to be more routinely used in a community setting, as opposed to higher dose-escalation strategies reported by many early multi-institutional series done at large academic centers [1, 12].

Here we outline as our primary endpoint, all-cause mortality reported as overall survival of the entire cohort measured from the time of therapy completion. Other endpoints include disease-free survival, local control, and correlations between local control and overall survival including tumor size, histology, tumor location, and treatment technique.

## Methods

Study approval was obtained from the North Shore-LIJ Health System (NSLIJHS), Monter Cancer Center Scientific Advisory Committee. A retrospective chart review protocol was further reviewed and approved by the NSLIJHS Institutional Review Board (IRB). Thereafter, a database of all patients diagnosed with lung cancer and treated with radiosurgery at North Shore Radiation Therapy from 2010 to 2012 was created to allow for at least a minimum of 2 years of follow-up. The following variables were collected during the analysis: date of treatment, patient age, tumor histology, tumor size, technique of treatment, tumor location, length of follow-up, toxicity, tumor control, and survival. IRB policies were followed regarding data deidentification.

### Radiotherapy technique and specifications

Due to the presence of a collaborative thoracic oncology program with prospective review, each patient was evaluated by a multidisciplinary team including a thoracic surgeon, interventional radiologist, medical and radiation oncologists. Patients were deemed medically inoperable based on objective criteria including a FEV1 < 1.5 L, pre-operative FEV1 < 40 % predicted value or DLCO <40 % of predicted value and at the final discretion of the surgeon. Many patients included in this study had fiducial markers placed by interventional radiology at the time of tumor biopsy unless they were deemed too high risk for this procedure. All patients were then simulated on a GE Lightspeed 64 slice 4D CT simulator and treated at North Shore Radiation Therapy/Cyberknife of Long Island. Target structures were delineated on pulmonary CT windows with the aid of PET fusion. Avidity (a sustained uptake value (SUV) of greater than 2) was included as part of gross tumor volume (GTV). For patients without fiducial markers or tumors not large enough to be seen and tracked using orthogonal radiographs, a 4D CT of the chest was used to account for tumor motion and create an internal target volume (ITV). The ITV was expanded 3–5 mm to account for set up error and arrive at the planning target volume (PTV). The total dose was prescribed to the 80 % isodose volume. The median biological BED used in this study was 105.6 Gy (range, 180–85.5 Gy_10_). Organs at risk and their respective tolerance doses used during treatment planning are shown in Table 1.

**Table 1:** Organ tolerance dose limits

Organ total dose volume		
Spinal cord	22 Gy Maximum	To any point
Esophagus	27 Gy maximum	To any point
Brachial plexus	24 Gy maximum	To any point
Aorta	45Gy maximum	To any point
Heart	30Gy maximum	To any point
Trachea and bronchus	30 Gy maximum	To any point
Chest wall/ ribs	30 Gy maximum^a^	To any point
Both lungs	20 Gy	<10 % of organ volume
Both Lungs	15 Gy	<35 % of organ volume
Skin	24 Gy maximum^a^	To any point

^a^considered for superficial tumors < 2 cm from chest wall

Three image guidance systems onboard the Cyberknife platform were used: the XSight Spine Tracking System, which relies on bony anatomy of the spine to locate and track tumors; the Synchrony Respiratory Motion Tracking System, which continuously synchronizes beam delivery with the motion of the target resulting from respiration by using external fiducial markers as a surrogate to track tumor motion; and the XSight Lung Tracking System, which tracks the soft tissue (tumor) target with respiration without the need for fiducial markers [13].

### Statistical analysis

Statistical objectives of this study included the estimation of median time until local progression, disease-free survival, all-cause mortality and acute toxicity. Survival was measured using the Kaplan-Meier method to characterize the progression of disease, local control and survival distributions. Median time to the event was estimated along with a 95 % confidence interval (CI). The Cox proportional hazards regression model was used to study the effects of multiple predictor variables (e.g. tumor size, stage, histology, patient age, tumor location (lobe), tracking method and biologic effective dose (BED)) on these three time-until-event outcomes. Descriptive statistics (proportions for categorical variables and means and standard deviations for continuous variables) were also calculated. Survival time was measured in months. Time until death was calculated from date of treatment completion to either date of event, or date of censoring (date of last follow-up), except for local failure at first follow-up scan post-treatment. In this case, time until death was measured from the date of first follow-up scan and not the date of treatment completion. For disease-free survival, failure was defined as any of the following: local failure, regional failure, distant failure, or locoregional failure. In cases where the event of death was not observed, the number of months until last follow-up was used and the subject’s vital status was classified as censored. The effects of categorical demographic, clinical, pathologic, and treatment variables were assessed using the log-rank test. Univariate Cox regression was used for continuous variables. A result was considered statistically significant if the p value was <0.05. To adjust for tied failure times, Efron’s method was used [14].

### End points and follow-up

Approximately three months after completing treatment, a follow-up radiologic examination was performed to determine the initial response to treatment. Two patients were followed by a non-contrast CT and the remaining with PET-CT scans. After the initial follow-up period, a chest CT or PET-CT scan was done to evaluate both tumor size and metabolism every 3 to 6 months for two years post treatment ––after which follow-up was done on an annual basis.

The definition of local control used in the current analysis was based on that used in RTOG 0236 [15]. Local failure was defined as meeting any one of the following criteria: (1) local tumor enlargement greater than 20 % of the gross tumor volume compared to the treatment planning CT-scan, (2) evidence of increasing metabolism using PET imaging, or (3) development of a new lesion in the involved lobe [15]. Regional failure was defined as a recurrence within a different ipsilateral lobe or any regional lymph node station including the bilateral hilar, mediastinal, scalene, or supraclavicular nodal stations as defined in the American Joint Committee on Cancer (AJCC) Cancer Staging Manual, 7^th^ edition [16]. Distant spread was defined as either radiographic evidence of a malignant pleural or pericardial effusion, pleural-based nodules, contralateral lung nodules, distant solid organ, CNS or osseous involvement.

## Results

A total of 67 medically inoperable patients with early and intermediate stage lung cancer were treated with stereotactic body radiotherapy (SBRT) between February 5, 2010 and November 4, 2013 (Table 2). Each patient had a single peripheral tumor defined as at least 2 cm from the trachea, carina and mainstem bronchi. One patient had limited follow-up, and was therefore included only in the overall survival analysis. Of the remaining 66 patients: 43 had local control, 11 failed locally, 5 regionally, 2 distantly and 5 locoregionally. Twenty-two of the 67 subjects eventually died. Twenty-three of 66 subjects had disease progression, and 6 had failed locally at the time of initial post-treatment evaluation. The mean follow-up was 24.5 months (range: 2.4-50.3 months). Among subjects who remained alive or were censored, the mean follow-up time was 28.6 months (range: 2.4-50.3 months).

**Table 2:** Patient characteristics (*N* = 67)

Age, years, median (range)		79 (60–92)
Sex	Male	*n* = 24 (36 %)
	Female	*n* = 43 (64 %)
Stage	IA	*n* = 52 (78 %)
	IB-III	*n* = 15 (22 %)
Location	Left Lower lobe	*n* = 16 (24 %)
	Left Upper Lobe	*n* = 16 (24 %)
	Right Lower Lobe	*n* = 12 (18 %)
	Right Upper Lobe	*n* = 23 (34 %)
Tumor volume	<2.5 cc	*n* = 17 (25 %)
	5-2.5 cc	*n* = 19 (29 %)
	5-10 cc	*n* = 7 (11 %)
	10-20 cc	*n* = 9 (13 %)
	>20 cc	*n* = 15 (22 %)
BED_10_^a^	Mean	107.8
	Median (Range)	105.6 (180–85.5)
Tracking	XSight-spine	*n* = 30 (45 %)
	XSight-lung	*n* = 3 (5 %)
	XSight-spine + Fiducials	*n* = 34 (50 %)
Histology	Adenocarcinoma	*n* = 30 (44 %)
	Squamous Cell Carcinoma	*n* = 14 (21 %)
	Non-small cell lung carcinoma-NOS^b^	*n* = 14 (21 %)
	Carcinoid	*n* = 3 (4 %)
	Sarcomatoid mesothelioma	*n* = 1 (2 %)
	Non-diagnostic biopsy	*n* = 6 (8 %)

^a^Biologic Effective Dose, Gy_10_ or α/β ratio = 10

^b^Not otherwise specified

### Overall survival based on local control status at first follow-up

All patients were imaged 1–6 months (median 3 months) after treatment. Due to the variability in how long after treatment a first follow-up scan was obtained, a sensitivity analysis was performed whereby the actual follow-up time from treatment to time of scan was adjusted for in a Cox regression model with local control status at time of first scan as the predictor of interest. The results from this analysis (data not shown) were similar to the Kaplan Meier estimate thus, only the Kaplan Meier results are reported as representative of local control status.

**Fig. 1:**
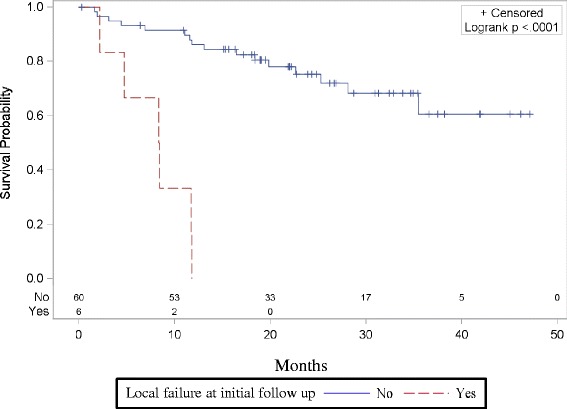
Achieving initial local control in previously untreated, medically inoperable patients with peripheral tumors targeted with SBRT is a predictor for improved survival. Comparing patients that achieved local control at first follow-up scan after SBRT was significantly associated with overall survival (OS) (*p* < 0.0001). OS at one year was 86.2 % (95 % CI, 74.3-92.9) in those achieving initial local control, whereas the one year survival probability for patients with local failure at first follow-up scan was 0.0 % (95 % CI, not estimable)

The achievement of local control at the time of initial follow-up was significantly associated with improved overall survival. Local control data were available for 66 patients. Among the 60 patients with local control determined at first follow-up, 16/60 died, whereas all 6 of the subjects who failed locally at their first follow-up scan succumbed to their disease. The estimated 1 year survival probability among patients achieving local control was 86.2 % (95 % CI, 74.3-92.9), whereas the one year survival probability for patients with local failure at first follow-up scan was 0.0 % (95 % CI, not estimable) (Fig. 1).

### Local control, disease-free and overall survival rates

**Fig. 2:**
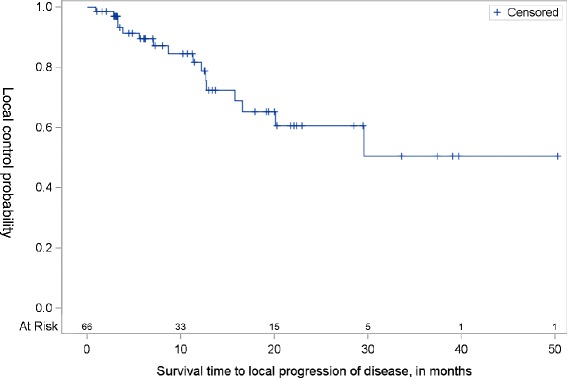
Local control in previously untreated, medically inoperable patients with peripheral tumors after being treated with Cyberknife SBRT. Local disease control at 12 months was 81.8 % (95 % CI, 67.3-90.3 %) and at 24 months was 60.6 % (95 % CI, 41.5- 75.2 %). The median time to local progression of disease was not reached

**Fig. 3:**
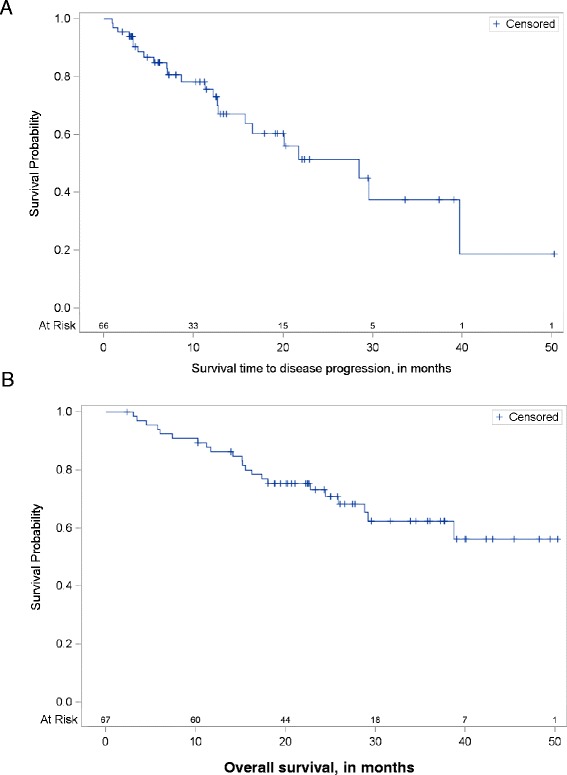
Disease free and overall survival in previously untreated patients with peripheral tumors deemed to be medically inoperable after being treated with Cyberknife SBRT.** a** The median progression-free survival was estimated to be 28.5 months (95 % CI: 15.8 months to not reached). The overall disease-free survival at 3 years was 37.5 % (95 % CI: 17.7- 57.4 %). **b** The overall survival at 3 years (36 months) was 62.4 % (95 % CI: 74.3-47.3 %). The median overall survival time was not reached

At 12 months, 81.8 % (95 % CI, 67.3-90.3) of patients maintained local control out of the total cohort. However, by 24 months only 60.6 % (95 % CI, 41.5- 75.2) of patients had local control although the median time to local progression of disease was never reached (Fig. 2). The overall disease-free survival at 3 years was 37.5 % (95 % CI: 17.7 to 57.4). Median disease-free survival was estimated to be 28.5 months (95 % CI: 15.8 months to not reached) (Fig. 3a). The overall survival at 3 years was 62.4 % (95 % CI: 74.3-47.3 %). The median overall survival time was not reached during the designated follow-up period (Fig. 3b).

### Comparative analysis of survival outcomes

A comparison of survival outcomes stratified by various patient and treatment-specific factors was not found to be statistically significant (using the log-rank test and *p* < 0.05). For example, analysis of time until disease progression with respect to gender (*p* = 0.8), stage (*p* = 0.16), pathology (*p* = 0.1), or tumor location/lobe involved (*p* = 0.15) did not reveal any significant differences (Additional file 1: Figure S1). Similarly, non-significant results were found in Cox regression analyses for BED_10_ (*p* = 0.43), age (*p* = 0.15), and tumor size (*p* = 0.66) with respect to disease-free survival (data not shown). Kaplan-Meier analysis for time until death was not found to be statistically significant among the following subgroups by univariate analyses: gender (*p* = 0.13), stage (*p* = 0.37), pathology (*p* = 0.77), and tumor location/lobe (*p* = 0.81) (Additional file 2: Figure S2). Results from a univariate Cox regression analysis did not provide statistical evidence to suggest a difference in overall survival according to BED_10_ (*p* = 0.08), age (*p* = 0.74), or tumor size (*p* = 0.29) (data not shown).

### Survival outcomes based on tracking method

**Fig. 4:**
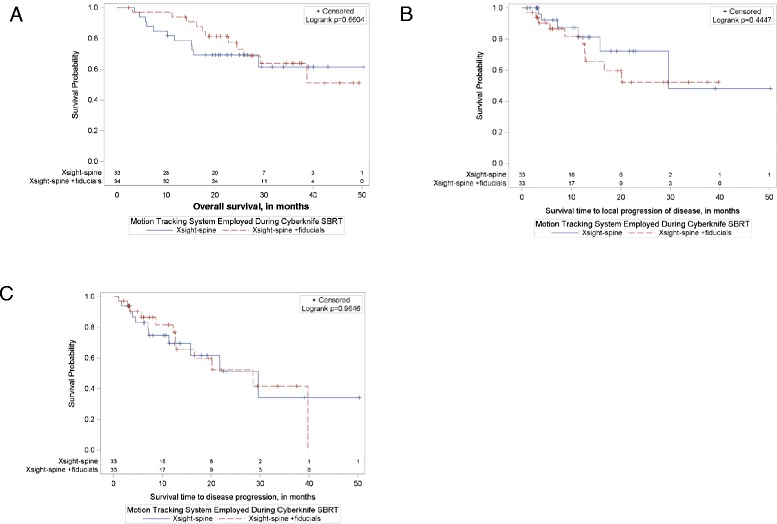
Comparing local control, disease free and overall survival rates in patients stratified by motion tracking system used during Cyberknife SBRT. Either a fiducial free (Xsight-spine/lung) or fiducial-based (Xsight-spine + fiducials) tracking system was used to localize and track tumors during each fraction of treatment. In the later technique, patients were initially aligned with Xsight-spine and then further adjusted through matching to intratumoral fiducials. **a** Overall Survival at 24 months was not statistically different in patients treated using Xsight-spine 69.2 % (95 % CL: 82.1-50.3 %) compared using Xsight-spine with fiducials 76.2 % (95 % CL: 88.6-58.0 %) (Logrank, *p* = 0.66) There was also no significant difference in **b** local control at 24 months for Xsight-spine alone 72.2 % (95 % CL: 94.8-49.6 %) vs. Xsight-spine with fiducials 52.3 % (95 % CL: 75.4-29.7 %) (Logrank, *p* = 0.44) or **c** disease free survival at 24 months when comparing Xsight-spine alone 51.4 % (95 % CL: 73.1-24.3 %) vs. Xsight-spine with fiducials, 52.3 % (95 % CL: 72.1-27.6 %) (Logrank, *p* = 0.97)

A comparison of overall survival in patients stratified by motion tracking and targeting system used during treatment was also done. Either non-invasive image guidance (XSight-spine/lung) alone or XSight-spine followed by matching to surgically implanted-intratumoral fiducials was used to localize and track tumors during each fraction of treatment. In the latter technique, tumors were initially aligned for treatment using the osseous spinal anatomy of each patient as a surrogate (XSight-spine) and then further adjusted through matching to radio-opaque fiducials within the tumor. In the event that tumor size was sufficient to visualize on orthogonal radiographs, XSight-lung localization was utilized (3 patients out of 67) after an initial matching to a portion of bony spine adjacent to the target. Survival was not found to be statistically different in patients treated using XSight-spine/lung compared to those treated with the aid of XSight-spine localization with fiducials (Logrank, *p* = 0.66). Additionally, there was no significant difference in local control or disease-free survival stratified by any tumor tracking method (Logrank, *p* = 0.44 and *p* = 0.97, respectively) (Fig. 4).

### Treatment-related toxicity

Toxicity was assessed immediately after treatment and again after 3 months of follow-up. Only four patients were documented as developing grade 1 fatigue. No grade 2 or higher toxicity was reported in this cohort at any time (data not shown).

## Discussion

In this study, medically inoperable patients with early or intermediate-stage peripheral lung tumors were treated with SBRT on a CyberKnife treatment platform to a range of biological effective doses (BED, 180–85.5 Gy_10_). The overall survival in our cohort was similar to that achieved in RTOG 0236 (62.4 % vs. 55.8 % at 36 months) [15]. Toxicity observed in this study, significant only for low grade fatigue, was also improved when compared to earlier studies—one of the first being the Indiana University phase I dose escalation and feasibility trial with 5 treatment-related deaths (pneumonia (*n* = 3), respiratory failure (*n* = 1), and hemoptysis (*n* = 1) occurring throughout the study period out of 70 patients treated with lung SBRT to a total dose of 60-66Gy in three fractions [12]. In light of these findings, we selected more conservative doses in an effort to balance the risk of toxicity with the efficacy of tumor control. In addition, we selected lower doses based on others’ observations reporting comparable 3 year local control rates above 80 % at 2 years using BEDs that approximate the range used in our cohort [17, 18]. In spite of these efforts, local control was lower than expected when compared to these historic controls.

We found that indeed, the local control rate at 24 months had decreased to 60.6 % compared to 91 % at three years seen in RTOG 0236 [15]. Although minor deviations in treatment planning may have contributed to this observation, the lower BEDs employed in this study are thought to be contributing factors compared to those used in earlier studies such as the Indiana University phase I dose escalation trial and RTOG 0236 where BEDs used were considerably higher— approaching 151.2 Gy_10_ once tissue heterogeneity corrections were applied [15, 12]. Furthermore, several others have reported that dose escalation correlated directly with significantly improved local control rates (Table 3) [6, 19–23]. It should also be emphasized that 22 % of the patients included in our cohort had stage IB to stage III disease and RTOG 0236 included patients with only T1-T2 tumors. Thus, it is possible that this may contribute to the lower local control rate seen in this study. Nevertheless, caution must be exercised when interpreting these results due to the limitations implicit within a retrospective study such as this. An additional limitation of this study is the relatively small sample size (*n* = 67 patients/treated lung tumors) which may explain the lack of statistical significance seen when performing a comparative analysis of survival outcomes to identify predictive factors (Additional file 1: Figure S1 and Additional file 2: Figure S2).

**Table 3:** Selected studies comparing local control as a function of BED

Author, year	Stage	Dose, BED10	Local control	P value
Van der Voort van Zyp et al. 2009 [19]	T1, *n* = 35; T2, *n* = 24	180 Gy	96 % at 2 years	n.s. *p* = 0.197
		112. 5 Gy	78 % at 2 years	
Onimaru et al. 2008 [20]	T1, *n* = 25; T2, *n* = 16	80 Gy	45 at 2 years	*p* = 0.0042
		105.6 Gy	89 % at 2 years	
Guckenberger et al. 2009 [21]	T1, *n* = 13; T2, *n* = 19; T3N0, *n* = 9	>100 Gy	89 % at 3 years	*p* = 0.0001
		<100 Gy	62 % at 3 years	
Bibault et al. 2012 [6]	T1, *n* = 31; T2, *n* = 20	≤150 Gy	70 % at 2 years	*p* = 0.006
		>150 Gy	100 % at 2 years	
Grills et al. 2012 [22]	T1, *n* = 318; T2, *n* = 67; T3, *n* = 10	<105 Gy	85 % at 2 years	*p* < 0.001
		>105 Gy	96 % at 2 years	
Onishi et al. 2007 [23]	Stage I, *n* = 257	>100 Gy	84 % at 3 years	*p* <0.01
		<100 Gy	37 % at 3 years	

As anticipated, patients that had achieved local control initially at the time of first follow-up had significantly improved overall survival compared with those that did not. These data support the notion that achieving initial local tumor control is of critical importance for survival.

Ablative therapies such as SBRT are now routinely designed without reliance on intra-target fiducial markers since the advent of sophisticated onboard-non-invasive image guidance systems [6, 13, 24]. Several phantom studies have demonstrated the ability to achieve sub-millimeter targeting with these systems [25]. However, evidence supporting the use of fiducial-free image guidance and localization systems is limited [6, 13, 25]. In this analysis, through a side-by-side comparison, we show that there is no difference statistically in survival outcome or tumor control metrics when stratifying patients by tracking technique used. Thus, non-invasive image-based tracking techniques appear to be safe and effective alternatives compared with traditional fiducial-based targeting and is in agreement with others’ reports [13, 18, 24, 25]. This has important implications in medically inoperable, frail patient populations with lung cancer that may not be able to easily tolerate such invasive procedures.

## Conclusions

This study supports the role of SBRT in the treatment paradigm for early-stage lung cancer in medically inoperable patients in order to gain local control of their disease, and doing so appears to be critical to achieving prolonged overall survival in this patient population. It provides evidence to justify the use of non-invasive tumor targeting during treatment administration over invasive fiducial-based targeting methods. Lastly, the importance of dose escalation is emphasized by our results especially with regard to the achievement of durable local tumor control. Nevertheless, further studies evaluating higher dose fractionation schemes are warranted to reach a comfortable equilibrium between better local control and low treatment-related morbidity.

## Additional files

Additional file 1: Figure S1. Comparison of disease free survival rates in patients stratified by gender, stage, pathology and location. The different Kaplan-Meier curves for time until disease progression were not found to be statistically significant (using the log-rank test and *p* < 0.05) among the following subgroups in univariate analysis: (A) Gender at 24 months comparing male patients, 49.3 % (95 % CL: 71.7-22.4 %) to female 54.3 % (95 % CL: 73.6-29.6 %), (*p* = 0.8), (B) Stage at 24 months comparing early stage patients with T1a disease 47.8 % (95 % CL: 64.9-28.5 %) to those with intermediate stage IB-III disease, 64.3 % (95 % CL: 90.2-15.2 %), (*p* = 0.16), (C) pathology at 24 months for patients with adenocarcinoma 41.4 % (95 % CL: 66.2-15.2 %), squamous cell carcinoma 30.3 % (95 % CL: 61.6-5.3 %), and unspecified non-small cell lung 64.3 % (95 % CL: 90.2-15.2 %), (*p* = 0.1) or (D) tumor location within the left lower lobe (LL), 50.1 % (95 % CL: 77.7-15.1 %), left upper lobe (LU), 73.7 % (95 % CL: 91.3-35.9 %) right lower lobe (RL), 28.3 % (95 % CL: 60.0-44.0 %), or right upper lobe (RU) 46.4 % (95 % CL: 75.2-12.6 %), (*p* = 0.15). Additional file 2: Figure S2. Comparison of overall survival rates in patients stratified by gender, stage, pathology and location. The different Kaplan-Meier curves for overall survival were not found to be statistically significant (using the log-rank test and *p* < 0.05) among the following subgroups in univariate analysis: (A) gender at 24 months comparing male patients, 87.1 % (95 % CL: 95.7-65.0 %) to female patients 65.9 % (95 % CL: 78.3-49.2 %) (*p* = 0.8), (B) stage at 24 months comparing early stage patients with T1a disease 69.7 % (95 % CL: 80.5-55.7 %) to those with intermediate stage IB-III disease, 86.7 % (95 % CL: 96.5-56.4 %) (*p* = 0.37), (C) pathology at 24 months for patients with adenocarcinoma 75.4 % (95 % CL: 87.5-55.2 %), squamous cell carcinoma 71.4 % (95 % CL: 88.2-40.6 %), and unspecified non-small cell lung 70.7 % (95 % CL: 87.9-39.4 %) (*p* = 0.77), or (D) tumor location within the left lower lobe (LL), 68.8 % (95 % CL: 85.6-40.5 %), left upper lobe (LU), 68.2 % (95 % CL: 85.4-39.5 %), right lower lobe (RL), 90.0 % (95 % CL: 98.5-47.3 %), or right upper lobe (RU), 73.9 % (95 % CL: 87.3-50.9 %) (*p* = 0.81).
