# Supplementary figures and images for: Medically inoperable peripheral lung cancer treated with stereotactic body radiation therapy
Source: Radiat Oncol. 2015 May 28;10:120. doi: 10.1186/s13014-015-0423-7 (PMC4461990; doi:10.1186/s13014-015-0423-7)

## Slide 1
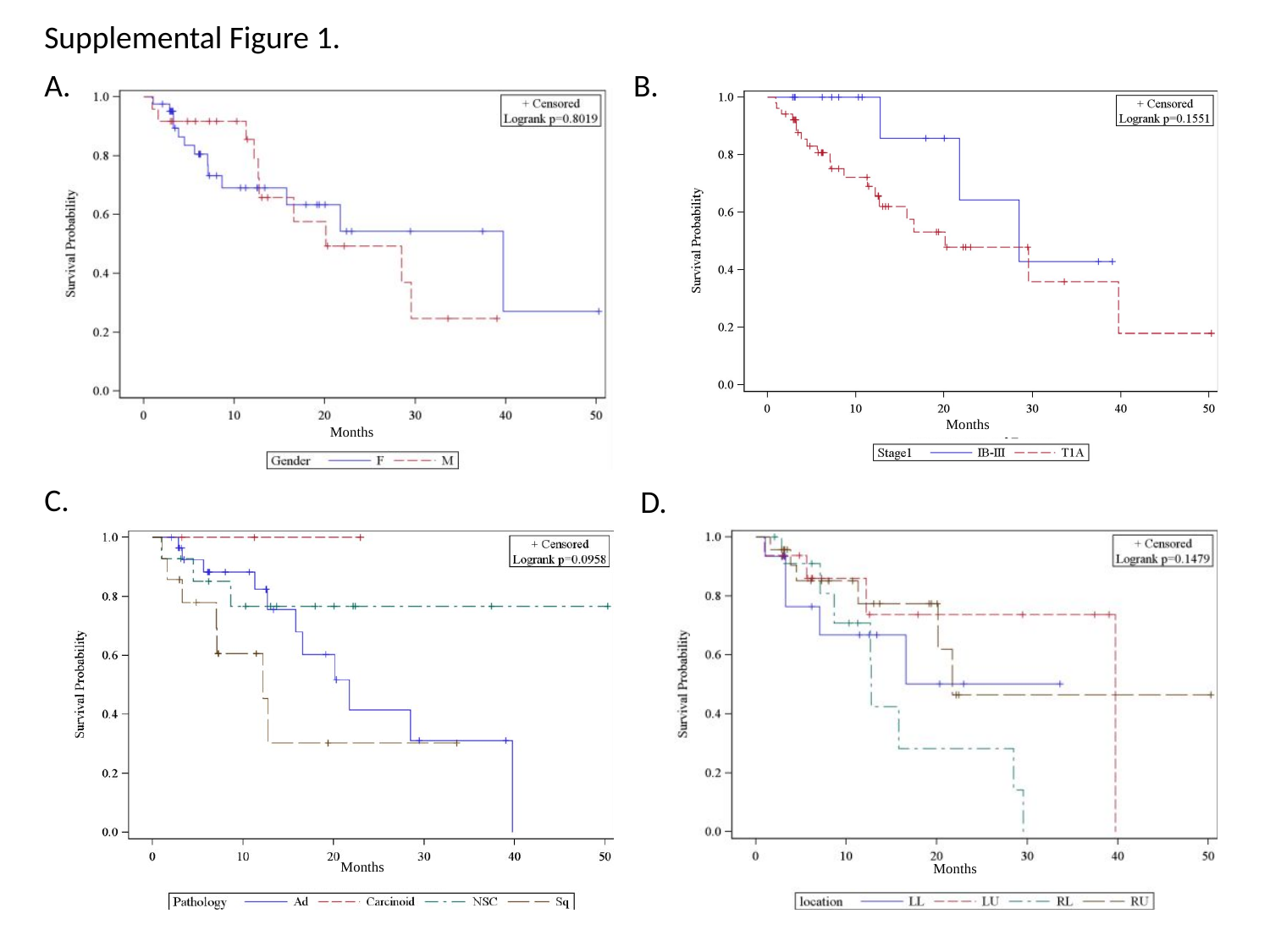

Supplemental Figure 1.
A.
B.
Months
Months
C.
D.
Months
Months

Supplement: Additional file 1: Figure S1. — Comparison of disease free survival rates in patients stratified by gender, stage, pathology and location. The different Kaplan-Meier curves for time until disease progression were not found to be statistically significant (using the log-rank test and p < 0.05) among the following subgroups in univariate analysis: (A) Gender at 24 months comparing male patients, 49.3 % (95 % CL: 71.7-22.4 %) to female 54.3 % (95 % CL: 73.6-29.6 %), (p = 0.8), (B) Stage at 24 months comparing early stage patients with T1a disease 47.8 % (95 % CL: 64.9-28.5 %) to those with intermediate stage IB-III disease, 64.3 % (95 % CL: 90.2-15.2 %), (p = 0.16), (C) pathology at 24 months for patients with adenocarcinoma 41.4 % (95 % CL: 66.2-15.2 %), squamous cell carcinoma 30.3 % (95 % CL: 61.6-5.3 %), and unspecified non-small cell lung 64.3 % (95 % CL: 90.2-15.2 %), (p = 0.1) or (D) tumor location within the left lower lobe (LL), 50.1 % (95 % CL: 77.7-15.1 %), left upper lobe (LU), 73.7 % (95 % CL: 91.3-35.9 %) right lower lobe (RL), 28.3 % (95 % CL: 60.0-44.0 %), or right upper lobe (RU) 46.4 % (95 % CL: 75.2-12.6 %), (p = 0.15). [file 13014_2015_423_MOESM1_ESM.pptx]

## Slide 1
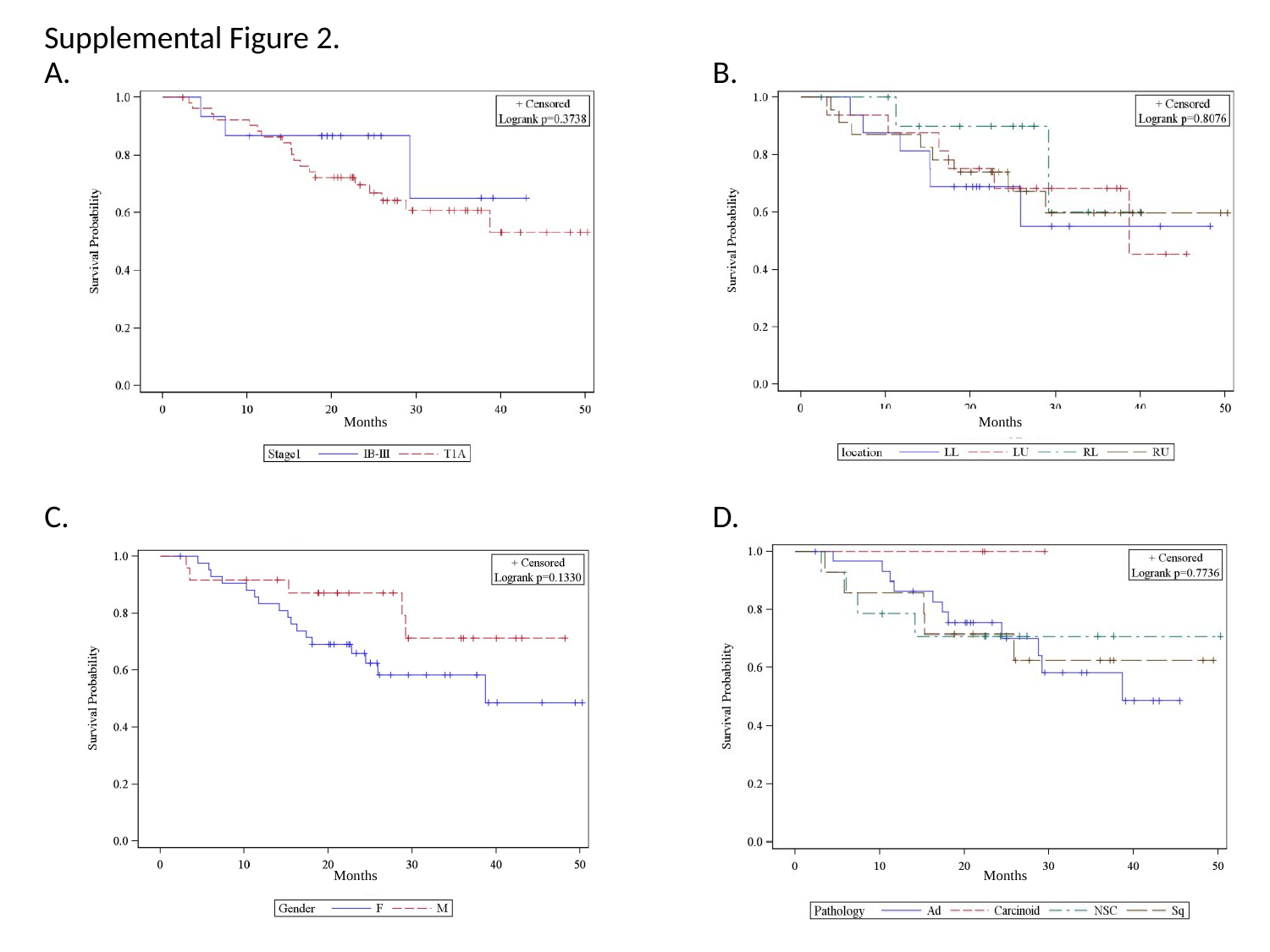

Supplemental Figure 2.
A.
B.
Months
Months
C.
D.
C.
Months
Months

Supplement: Additional file 2: Figure S2. — Comparison of overall survival rates in patients stratified by gender, stage, pathology and location. The different Kaplan-Meier curves for overall survival were not found to be statistically significant (using the log-rank test and p < 0.05) among the following subgroups in univariate analysis: (A) gender at 24 months comparing male patients, 87.1 % (95 % CL: 95.7-65.0 %) to female patients 65.9 % (95 % CL: 78.3-49.2 %) (p = 0.8), (B) stage at 24 months comparing early stage patients with T1a disease 69.7 % (95 % CL: 80.5-55.7 %) to those with intermediate stage IB-III disease, 86.7 % (95 % CL: 96.5-56.4 %) (p = 0.37), (C) pathology at 24 months for patients with adenocarcinoma 75.4 % (95 % CL: 87.5-55.2 %), squamous cell carcinoma 71.4 % (95 % CL: 88.2-40.6 %), and unspecified non-small cell lung 70.7 % (95 % CL: 87.9-39.4 %) (p = 0.77), or (D) tumor location within the left lower lobe (LL), 68.8 % (95 % CL: 85.6-40.5 %), left upper lobe (LU), 68.2 % (95 % CL: 85.4-39.5 %), right lower lobe (RL), 90.0 % (95 % CL: 98.5-47.3 %), or right upper lobe (RU), 73.9 % (95 % CL: 87.3-50.9 %) (p = 0.81). [file 13014_2015_423_MOESM2_ESM.pptx]
